# Prediction of Heterodimeric Protein Complexes from Weighted Protein-Protein Interaction Networks Using Novel Features and Kernel Functions

**DOI:** 10.1371/journal.pone.0065265

**Published:** 2013-06-11

**Authors:** Peiying Ruan, Morihiro Hayashida, Osamu Maruyama, Tatsuya Akutsu

**Affiliations:** 1 Bioinformatics Center, Institute for Chemical Research, Kyoto University, Gokasho, Uji, Kyoto, Japan; 2 Institute of Mathematics for Industry, Kyushu University, 744 Motooka, Nishi-ku, Fukuoka, Japan; Instituto de Tecnologica Química e Biológica, UNL, Portugal

## Abstract

Since many proteins express their functional activity by interacting with other proteins and forming protein complexes, it is very useful to identify sets of proteins that form complexes. For that purpose, many prediction methods for protein complexes from protein-protein interactions have been developed such as MCL, MCODE, RNSC, PCP, RRW, and NWE. These methods have dealt with only complexes with size of more than three because the methods often are based on some density of subgraphs. However, heterodimeric protein complexes that consist of two distinct proteins occupy a large part according to several comprehensive databases of known complexes. In this paper, we propose several feature space mappings from protein-protein interaction data, in which each interaction is weighted based on reliability. Furthermore, we make use of prior knowledge on protein domains to develop feature space mappings, domain composition kernel and its combination kernel with our proposed features. We perform ten-fold cross-validation computational experiments. These results suggest that our proposed kernel considerably outperforms the naive Bayes-based method, which is the best existing method for predicting heterodimeric protein complexes.

## Introduction

Protein complexes play crucial roles in a variety of biological processes, such as ribosomes for protein biosynthesis, molecular transmission and evolution of interactions between proteins. In fact, many proteins come to be functional only after they interact with their specific partners and are assembled into protein complexes. Hence, much effort has been made for predicting protein complexes from protein-protein interaction (PPI) networks [1]–[6] in bioinformatics. The Markov Cluster (MCL) algorithm [7] iteratively generates a matrix, called Markov matrix, in which each row (each column) corresponds to a protein and each element represents the relationship between two proteins. Then, MCL extracts clusters from the matrix. This algorithm is efficient also for large-scale networks because Markov matrices are calculated by matrix multiplication and exponentiation of its individual elements. The Molecular Complex Detection (MCODE) algorithm [8] gives a weight to each vertex by using a modified clustering coefficient, which is defined as edge density in a subset of neighboring vertices and the originating vertex. Then, it finds densely connected regions of molecular interaction networks based on the weighted vertices. The Restricted Neighborhood Search Clustering (RNSC) algorithm [9] separates the set of vertices into clusters by searching locally in a randomized fashion based on a cost function. After that, the clusters will be filtered according to the cluster size, density and functional homogeneity. The Protein Complex Prediction (PCP) algorithm [10] finds maximal cliques within PPI networks modified by using the functional similarity weight (FS-Weight) based on indirect interactions, and merges their cliques. These methods are intended for detecting dense subgraphs in a PPI network. Hence, they cannot find a protein complex with size two because the density is always 1.0 and the subgraph (i.e., an edge) itself is a clique even if two proteins that interact with each other do not form a complex. In addition, it is considered that any overlap rate of a predicted protein complex to a small known complex is more likely to be by chance than the same overlap rate to a larger known complex as pointed out in [11]. Most prediction methods have been evaluated for protein complexes with larger size than three excluding complexes with small sizes.

However, the majority of known protein complexes are heterodimeric protein complexes. CYC2008 [12], which is a comprehensive catalogue of 408 manually curated yeast protein complexes reliably supported by small-scale experiments, includes 172 (42%) heterodimeric protein complexes. Besides, MIPS protein complex catalog [13], which provides detailed information involved protein sequences on whole-genome analysis [14]–[16], contains 64 (29%) heterodimeric protein complexes excluding complexes obtained from high-throughput experiments. Hence, it is necessary to develop another method for predicting smaller complexes. Qi et al. proposed a method using a supervised Bayesian classifier [17] that has good performance for predicting protein complexes of middle sizes. The method still does not work well for heterodimeric protein complexes because they used several features based on graph density and degree statistics. There are some approaches based on random walks on PPI networks. The Repeated Random Walks (RRW) method [18] repeatedly expands a focused cluster of proteins depending on the steady state probability of random walks with restarts from the cluster whose proteins are equally weighted. The Node-Weighted Expansion (NWE) method [19] is an extension of RRW. NWE restarts from the cluster whose proteins are weighted by the sum of the edge weights of the physical interactions with neighboring proteins, where the edge weights are obtained from the WI-PHI database [1]. Then, Maruyama [11] proposed an approach based on a naive Bayes classifier using heterogeneous genomic data for predicting heterodimeric protein complexes with features involved with protein-protein interaction data, gene expression data, and gene ontology annotations. This method outperforms other existing prediction methods, MCL, MCODE, RRW, and NWE, in F-measure for heterodimers [11] although these methods are not supervised.

To further improve the prediction accuracy for heterodimeric protein complexes, we propose a method using *C*-Support Vector Classification (*C*-SVC) with several features based on protein-protein interaction weights that are considered as reliability of interactions between proteins. The idea behind the design of feature space mappings is, for example, that the neighboring weights of a heterodimeric complex tend to be smaller than the weight inside of the complex. In addition to features based on weights, we propose feature space mappings based on the numbers of protein domains because those are considered to be functional and structural units in proteins. Furthermore, we propose a domain composition kernel based on the idea that two proteins having the same composition of domains as a heterodimeric protein complex would also form a heterodimer. We perform ten-fold cross validation, and calculate the average F-measures. The results suggest that our proposed kernel considerably outperforms the naive Bayes-based method, which is the best existing method.

## Methods

The problem we address in this study is stated as follows: Given a network of protein-protein interactions, where interactions are weighted, determine whether or not two interacting distinct proteins form a protein complex with size exactly two. A network of protein-protein interactions can be considered as a graph, where vertices represent proteins and edges represent protein interactions. Let *G*(*V*, *E*) be an undirected graph with a set *V* of vertices and a set *E* of edges, where the weight of each edge 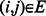 is denoted by *w_ij_* and represents reliability and strength of the interaction related with the edge. Actually, we use the WI-PHI database [1] as edge weights, which is derived from heterogeneous data sources, and was used in previous studies [11], [18], [19]. In this section, we propose several features for predicting heterodimeric protein complexes, a novel kernel matrix based on protein domain composition, and the combination kernel.

### Feature Space Mapping Based on Interaction Weights

We propose simple feature space mappings based on weights of interactions, which are regarded to be reliabilities and strengths for protein-protein interactions as shown in Table 1. The basic idea for designing features is as follows. The reliability of the interaction in a heterodimeric complex should be high. In addition, the reliability of the interaction between a protein contained in a complex and a protein not contained in the complex should be low. These features are not only applied to *C*-SVC through linear kernels but are transformed to other kernel matrices using extended diffusion and label sequence kernels.

**Table 1:** Feature space mapping from two interacting proteins *P_i_*, *P_j_* and neighbors.

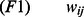
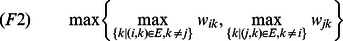
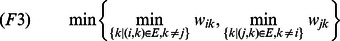
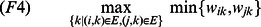
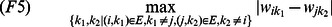
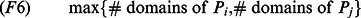
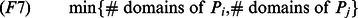

**Figure 1:**
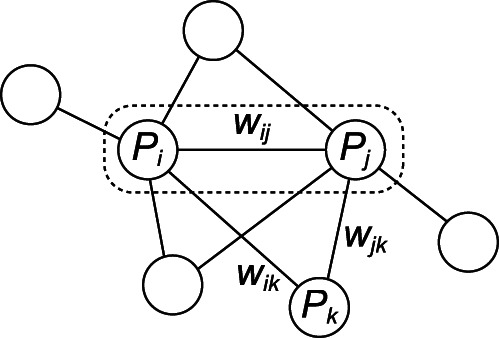
Example of a subgraph with an interacting protein pair and their neighboring proteins. 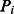
 and 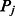 denote focusing interacting proteins shown in the dashed rectangle. 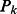 is a neighboring protein. 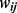 denotes the weight of the interaction between 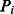 and 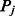.

Consider two interacting proteins *P_i_* and *P_j_* corresponding to an input. Figure 1 shows an example of a subgraph with *P_i_*, *P_j_*, and their neighboring proteins *P_k_* such that 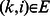 or 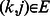, where interactions between these proteins are shown as edges. One feature is the weight 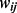 between proteins 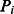 and 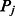, denoted by (F1), because the proteins in a heterodimeric protein complex should interact with each other and the weight 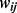 should be large.

However, even if 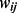 is large, the proteins could be included in a complex with size larger than two. Hence, we consider the weights of interactions with the neighboring proteins 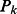. Since the neighboring weights of a heterodimeric complex tend to be smaller than the weight inside of the complex, we introduce the maximum of the neighboring weights denoted by (F2) as a feature.

In contrast, if the neighboring weights are larger than the weight 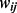, we can estimate that the proteins 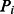 and 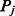 would not form a complex but neighboring proteins and either 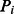 or 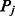 would form some complex. Thus, we introduce the minimum of the neighboring weights denoted by (F3).

Even if the maximum of the neighboring weights (F2) is large enough, the proteins 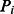 and 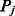 as well as 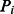 and 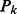 or  and 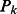 may form a heterodimeric complex. Consider the case that a protein 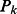 interacts with both of 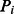 and 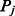. If two weights 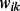 and 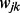 are large, these proteins 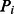, 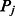 and 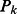 are likely to form a complex. Besides, if 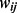 is smaller than 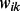 and 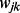, 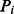, 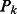 and 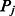, 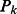 independently can form a heterodimeric complex. For this reason, we introduce the maximum of smaller weights denoted by (F4).

In the discussion so far, we dealt only with the value of weights. However, differences between weights are also important for discriminating heterodimeric complexes. Hence, we introduce the maximum of differences between the neighboring weights denoted by (F5).

For prediction of complexes, biological knowledge for proteins is helpful. We use protein domains that are parts of proteins known as structural and functional units. Ozawa et al. introduced the domain structural constraint that one domain interacts with at most one other domain for verifying protein complexes [20]. The constraint excludes extra proteins from a set of proteins that is a candidate complex by validating possible interactions between domains. This means that extra domains cause interactions with other proteins and the actual number of proteins contained in the complex may be greater than that in the candidate set of proteins. Since two proteins with small numbers of domains tend to form a heterodimeric complex, we introduce the maximum of the numbers of domains contained in 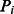 and 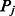 denoted by (F6). In contrast, we introduce the minimum of the numbers of domains contained in 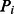 and 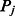 denoted by (F7) because proteins with large numbers of domains tend to form complexes with large sizes.

### Domain Composition Kernel

In the previous section, we introduced several feature space mappings from an example, that is, a pair of proteins. Kernel functions can incorporate prior knowledge. If a set of proteins has the same composition of domains as a known complex, it is highly expected that the set forms a complex. On the basis of this idea, we propose domain composition kernel for candidate complexes 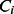 and 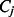 with size 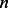 (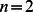 in this paper), in which 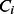 and 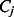 are regarded as sets of proteins, 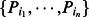 and 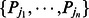, respectively. Then, we define equivalence 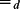 between two proteins 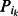 and 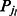 as 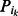 consists of the same domains of 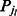, where the number of each domain must also be the same between the proteins. Furthermore, we define equivalence 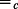 between two sets of proteins 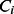 and 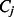 using 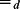 by
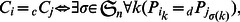
(1)where 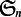 denotes the symmetric group of degree 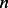 on the set 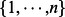 (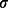 is a permutation of 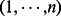). For example, in the case of 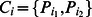 and 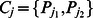, 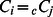 if 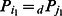 and 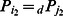 or 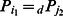 and 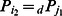, whereas it is not necessary that 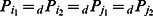.

Then, we propose domain composition kernel 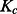 by
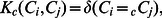
(2)where 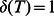 if 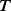 holds, otherwise 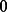. It should be noted that our kernel is different from pairwise kernels for protein pairs proposed in [21]. Their kernel is defined as 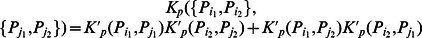 for predicting protein-protein interactions, where 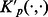 is called ‘genomic kernel’ and operates on individual genes or proteins. In the case of 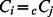, that is, 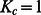, 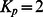 if 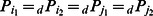, otherwise 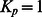, where 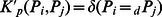. In addition, their pairwise kernels allow extra domains in a candidate complex because the domains do not prevent two proteins to interact with each other.

We can prove that 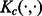 is a kernel.

**Theorem 1** 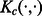 defined by Eq. (2) is a positive semidefinite kernel.

*Proof)* We show that the Gram matrix ***K*** for a set of candidate complexes 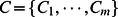 is positive semidefinite. The binary relation 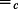 on the candidate set is an equivalence relation because for all 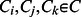, 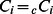 (reflexivity), if 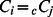 then 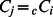 (symmetry), if 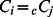 and 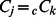 then 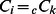 (transitivity). Then, the relation 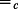 partitions 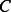 into 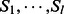, and we have for any vector 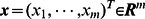


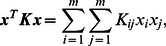
(3)

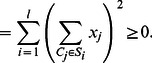
(4)It should be noted that 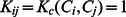 if 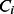 and 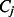 are classified in the same set, otherwise 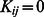. Consequently, ***K*** is positive semidefinite, and 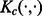 is a valid kernel. 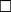.

In addition, for the purpose of predicting whether or not two interacting proteins form a heterodimeric complex, we combine some feature space mapping 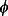 in Table 1 with the domain composition kernel by
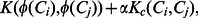
(5)where 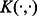 is any kernel for real-valued vectors, and 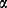 is a positive constant. In this paper, we use the linear kernel for 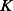, that is, 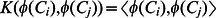.

## Computational Experiments

### Data and Implementation

To perform computational experiments, we needed protein-protein interaction data with weights and protein complex data. We used the WI-PHI database [1] including 49607 protein pairs except self interactions as weighted protein-protein interaction data, where the actual file name was ‘pro200600448_3_s.csv’ at the supporting information web page of http://www.wiley-vch.de/contents/jc_2120/2007/pro200600448_s.html. The weights of interactions were calculated as follows. They constructed the literature-curated physical interaction (LCPH) dataset using several databases such as BioGRID [2], MINT [3], and BIND [4], and high-throughput yeast two-hybrid data by Ito [22] and Uetz [23]. To evaluate high-throughput data, they constructed a benchmark dataset having interactions supported by two independent methods from LCPH-LS, which was a low-throughput dataset in LCPH, and calculated a log-likelihood score (LLS) to each dataset except LCPH-LS. For each interaction, the weight was calculated by multiplying the socioaffinity (SA) indices [15] and the LLSs from different datasets, where the SA index measures the log-odds score of the number of times two proteins are observed to interact to the expected value from their frequency in the dataset.

**Figure 2:**
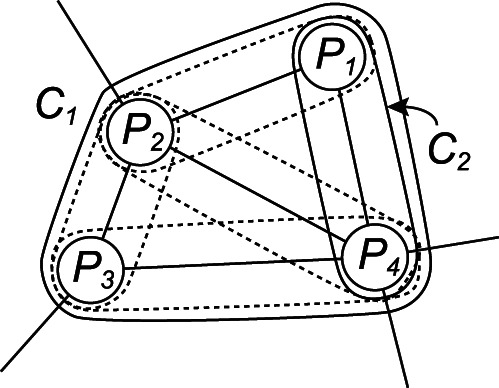
Illustration of the selection of negative examples from complexes with size more than two. Complex 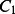 consists of four proteins 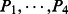, whereas heterodimeric complex 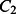 consists of 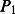 and 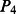. Edges represent protein-protein interactions. According to this figure, four sets of two proteins, 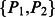, 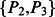, 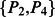, and 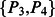 are selected as negative examples. The set of two proteins 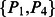 is removed from the dataset. Each pair of two proteins surrounded by a dashed curve corresponds to a negative example.

To compare our method with the naive Bayes-based method proposed by Maruyama [11], we prepared the same dataset as in the paper [11] from CYC2008 protein complex database [12], which is available at http://wodaklab.org/cyc2008/resources/CYC2008_complex.tab. In the dataset, a positive example was restricted to a pair of proteins that is included as a PPI in WI-PHI and is not a proper subset of any other complex in CYC2008. Thus, we used 152 heterodimeric protein complexes contained in CYC2008 as positive examples, and selected 5345 negative examples from interacting protein pairs in the CYC2008 complexes with size more than two, where positive examples were excluded. Figure 2 shows an example of complexes 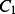 and 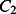 consisting of four proteins 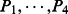 and two proteins 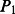 and 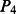, respectively. According to this figure, four sets of two proteins, 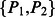, 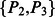, 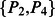, and 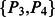 are selected as negative examples, where each interaction between two proteins is confirmed to be included in WI-PHI. The set of two proteins 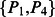 is removed from the dataset. Since negative examples selected in this way are more difficult to be correctly predicted than randomly selected ones, this dataset is considered to be useful for the evaluation.

#### 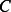-Support Vector Classification (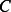-SVC) for unbalanced data

Since the numbers of positive and negative examples of the dataset used in this paper were very unbalanced, we used the extension of 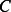-Support Vector Classification (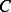-SVC) described in [24], [25]. The extended 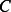-SVC solves the following optimization problem given input feature vectors 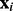 and the corresponding classes 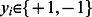. 
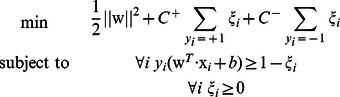
where 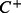 and 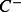 are regularization parameters for positive and negative classes, respectively, and in the usual 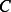-SVC, 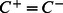.

We used ‘libsvm’ (version 3.11) [26] as an implementation of 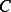-SVC for unbalanced data.

#### Performance measure

To evaluate the performance of our method, we used precision, recall and F-measure, which are defined by
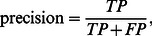
(6)

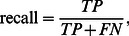
(7)

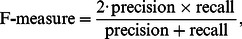
(8)where 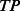, 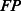, and 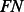 denote the numbers of true positive, false positive, and false negative examples, respectively. Precision means the rate of correctly predicted positive examples to examples predicted as positive, and recall means the rate of correctly predicted positive examples to all positive examples. For evaluation of binary predictors, it is not sufficient to calculate only either the precision or the recall, and thus we used F-measure of their harmonic mean.

## Results

To evaluate our method, we used several sets of our proposed features, (F1–5), (F1–6), (F1–5,7), and (F1–7). For example, (F1–5) means that we use a feature vector consisting of five values calculated by (F1), (F2), 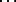, (F5) as shown in Table 1. Then, we calculated the combination kernel with the domain composition kernel as shown in Eq.(5), and employed 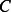-SVC with varying mixing parameter 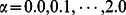 and regularization parameters 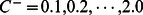, 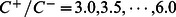. For each case, we performed 10-fold cross-validation using our combination kernel, and took the average of precision, recall, and F-measure in the same way as in [11].

**Figure 3:**
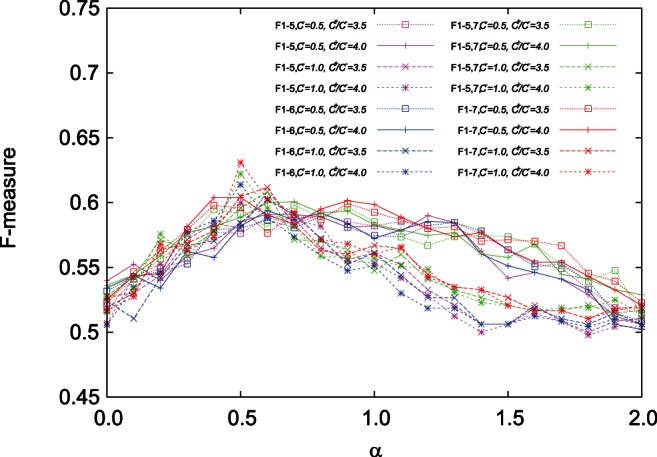
Result on the average F-measures using four sets of features and the domain composition kernel with 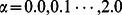. 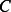
-SVC was employed with regularization parameters, 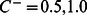, 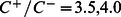. As sets of features, (F1–5), (F1–6), (F1–5,7), and (F1–7) shown in Table 1 were used.

Figure 3 shows the results on the average F-measures using four sets of features, (F1–5), (F1–6), (F1–5,7), (F1–7), and the domain composition kernel for the cases of 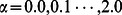, 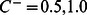, 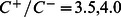 (see Fig. S1 for more cases of 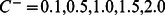 and 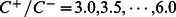). We can see from these figures that the average F-measures during 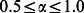 were about 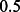 to 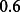 and were better than that of 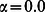 in each case. It means that the domain composition kernel enhanced the prediction accuracy comparing with only features. Furthermore, features (F1–7) tended to have better average F-measures than other sets of features.

Table 2 shows the results on the average precision, recall, and F-measure using our features and domain composition kernel in the best average F-measures case for each set of features. It also shows the results by the naive Bayes-based method [11], which is the best existing method for heterodimeric complex prediction, MCL [7], MCODE [8], RRW [18], and NWE [19]. (B1), (B2:CC), …, (B6) indicate the features used in the naive Bayes-based method (shown also in Table 3). These existing methods were executed using default parameters except the option of the minimum size of predicted complexes, which was set to be two if possible. For sets of features (F1–5), (F1–6), (F1–5,7), and (F1–7), the average F-measures in the cases of 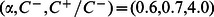, 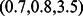, 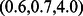, and 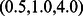 were best, respectively. In particular, the average F-measure for (F1–7) using 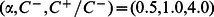 was best among all the cases, and was much better than that by the naive Bayes-based method. We investigated which feature most contributed to the prediction accuracy. The discriminant function for SVM with linear kernel can be represented as 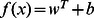. Here we suppose that elements 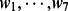 of ***w*** are the coefficients of the corresponding features (F1),(F7), respectively. If each element of ***x*** is normalized, it can be considered that features with the largest absolute value of 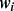 are effective for the discrimination in the seven features. We calculated the coefficients and averages of the feature values using 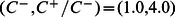 and the dataset with 152 positive and 5345 negative examples. Thus, we had the coefficients 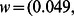 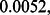 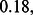 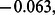 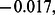 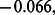 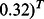, 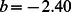, and the averages 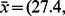 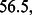 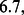 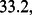 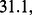 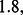 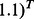. Then, 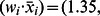 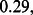 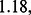 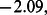 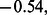 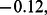 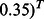, and it was (F4),(F1),(F3),(F5),(F7),(F2),(F6) in descending order of 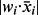. We can see that (F4) was most effective, and worked on the discrimination negatively, whereas (F6) was least effective, in fact, the decrease of the average F-measure by removal of (F6) from (F1–7) was small as shown in Table 2. It should be noted that this result does not necessarily mean that supervised methods such as the naive Bayes-based method and our proposed method are always better than unsupervised methods such as MCL and MCODE because unsupervised methods were evaluated using the whole PPI data whereas supervised methods were trained and evaluated via cross validation using a part of PPI data. Therefore, unsupervised methods may work better in other situations.

**Table 2:** Result on the average precision, recall, and F-measure using our features and domain composition kernel in the best average F-measure case for each set of features.

method	features	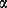	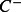	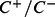	precision	recall	F-measure
Our combination kernel	F1–5	0.6	0.7	4.0	0.586	0.659	0.620
	F1–6	0.7	0.8	3.5	0.566	0.677	0.616
	F1–5,7	0.6	0.7	4.0	0.592	0.667	0.627
	F1–7	0.5	1.0	4.0	0.618	0.644	**0.631**
naive Bayes [11]	B1, B2:CC	–			0.24	0.44	0.31
	B1–6	–			0.17	0.65	0.27
MCL [7]	–				0.017	0.023	0.020
MCODE [8]	–				0	0	–
RRW [18]	–				0.030	0.32	0.055
NWE [19]	–				0.035	0.33	0.063

As sets of features, (F1–5), (F1–6), (F1–5,7), and (F1–7) shown in Table 1 were used. The results by the naive Bayes-based method [11], MCL [7], MCODE [28], RRW [18], and NWE [19] are also shown, where the experiments for these methods were performed by [11]. (B1), (B2:CC),, (B6) indicate the features by [11] (shown also in Table 3).

**Table 3:** Feature space mapping from two interacting proteins 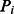, 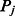 in the naive Bayes-based method [11].

(B1)	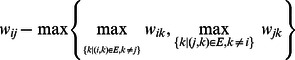
(B2:X)	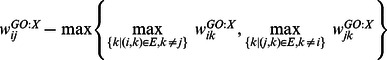 , where 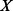 represents
	an ontology among biological process (BP), cellular component (CC) and molecular
	function (MF) of Gene Ontology [27], and is also regarded to be the set of the terms;
	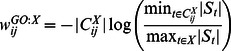 , where 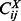 is the set of all terms in 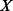 annotating
	both 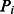 and 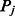 , and 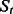 is the set of proteins annotated by term 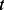 .
(B3)	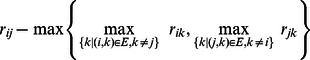 , where 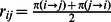 and
	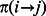 is the stationary probability from 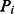 to 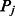 by a random walk with restarts
	at 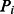 (RRW [18]).
(B4)	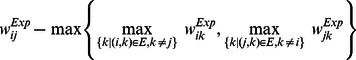 , where 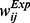 is the Pearson
	correlation coefficient between the two genes producing 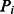 and 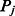 , respectively, over
	some gene expression profiles.
(B5)	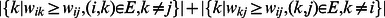
(B6)	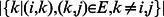

**Figure 4:**
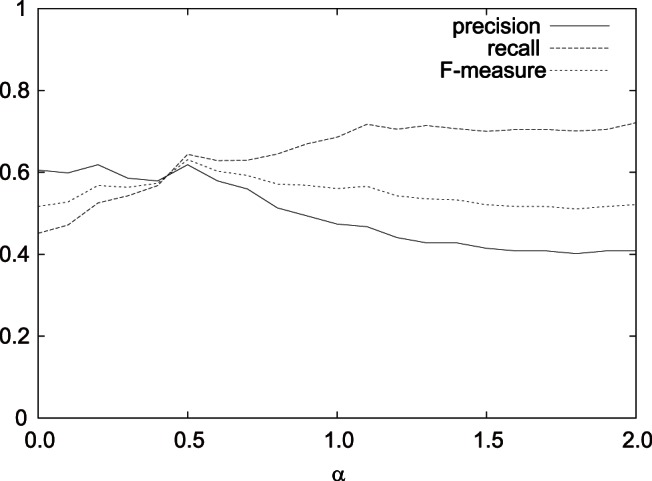
Result on the average precision, recall, and F-measure with varying 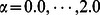 in the best case using features (F1–7).

**Figure 5:**
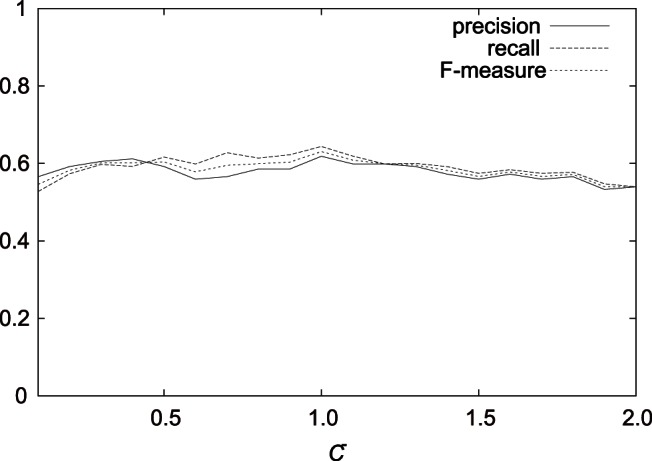
Result on the average precision, recall, and F-measure with varying 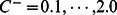 in the best case using features (F1–7).

**Figure 6:**
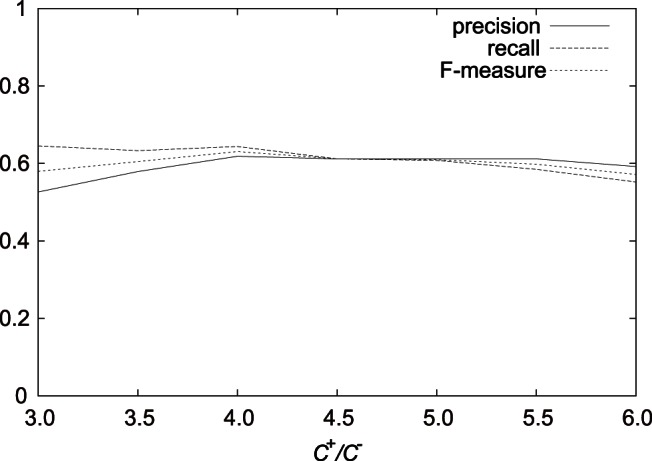
Result on the average precision, recall, and F-measure with varying 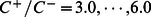 in the best case using features (F1–7).

Figures 4, 5, and 6 show the results on the average precision, recall, and F-measure with varying 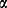, 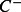, and 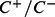, respectively, in the case of 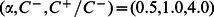 using features (F1-7). We can see that in the examined range, the average F-measures did not largely fluctuated.

In addition, we performed another experiment to validate our method for the rest PPIs, that is, we used 152 positive and 5345 negative examples as training data, and used the rest, 44110 examples as test data. Then, we obtained the prediction accuracy of 98.7% (43554/44110) using the combination kernel with (F1–7) and 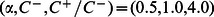. These results suggest that our proposed kernel successfully predicted heterodimeric protein complexes and outperforms the naive Bayes-based method.

### Conclusions

We proposed several feature space mappings using weights of protein-protein interactions for predicting heterodimeric protein complexes. In addition, we proposed the domain composition kernel based on the idea that two proteins having the same composition of domains as a heterodimeric protein complex would also form a heterodimer, and proved that the domain composition kernel is actually a kernel function. To validate our proposed method, we performed ten-fold cross-validation computational experiments for the combination kernel of the domain composition kernel with the linear kernel using several sets of features. The results suggest that our proposed kernel considerably outperforms the naive Bayes-based method, which is the best existing method, even in the case using only feature space mappings (F1–5) from weights of protein-protein interactions, that is, (F6,7) was not used and the mixing parameter 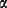 is 0 although our proposed method is limited to prediction of heterodimeric protein complexes.

An important contribution in this paper is that we have shown that heterodimeric protein complexes are able to be successfully predicted using only information on weights of protein-protein interactions. Furthermore, we indicated that the use of protein domain information enhances the prediction accuracy.

There is some possibility to further improve the prediction accuracy. For instance, we can develop some kernels on protein domains using protein amino acid sequences and multiple sequence alignments. In addition, we can add new features based on other biological knowledge.

We used the *C*-SVC classifier, which is a variant of support vector machines, because the numbers of positive and negative examples were not balanced. It is interesting future work to develop more robust methods against unbalanced data for classifying heterodimeric protein complexes.

## Supporting Information

Figure S1
**Result on the average F-measures using four sets of features and the domain composition kernel with 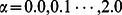.** ***C*** **-SVC was employed with regularization parameters, 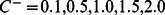, 
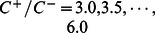
.** As sets of features, (F1–5), (F1–6), (F1–5,7), and (F1–7) shown in Table 1 were used.(EPS)

Figure S2
**Result on the average F-measures using four sets of features and the domain composition kernel represented by Eq. (S1) with 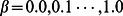.** ***C*** **-SVC was employed with regularization parameters, 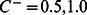, 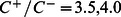.** As sets of features, (F1–5), (F1–6), (F1–5,7), and (F1–7) were used.(EPS)

Table S1 **Result on the average precision, recall, and F-measure using our combination kernel represented by Eq. (S1) in the best average F-measure case for each set of features.** As sets of features, (F1–5), (F1–6), (F1–5,7), and (F1–7) were used.
(PDF)

Text S1 **Results on our kernel by another combination.**
(PDF)
