# Supplementary material for: Prediction of Heterodimeric Protein Complexes from Weighted Protein-Protein Interaction Networks Using Novel Features and Kernel Functions
Source: PLoS One. 2013 Jun 11;8(6):e65265. doi: 10.1371/journal.pone.0065265 (PMC3679142; doi:10.1371/journal.pone.0065265)
Supplement: Table S1 — Result on the average precision, recall, and F-measure using our combination kernel represented by Eq. (S1) in the best average F-measure case for each set of features. As sets of features, (F1–5), (F1–6), (F1–5,7), and (F1–7) were used. (PDF) [file pone.0065265.s003.pdf]

features	β	C^-	C^+/C^-	precision	recall	F-measure
F1-5	0.4	1.0	3.5	0.5461	0.6860	0.6081
F1-6	0.7	0.5	3.5	0.5395	0.6777	0.6007
F1-5,7	0.7	0.5	3.5	0.5395	0.6833	0.6029
F1-7	0.4	0.5	4.0	0.6184	0.5987	0.6084
