# Supplementary material for: Prediction of Heterodimeric Protein Complexes from Weighted Protein-Protein Interaction Networks Using Novel Features and Kernel Functions
Source: PLoS One. 2013 Jun 11;8(6):e65265. doi: 10.1371/journal.pone.0065265 (PMC3679142; doi:10.1371/journal.pone.0065265)
Supplement: Text S1 — Results on our kernel by another combination. (PDF) [file pone.0065265.s004.pdf]

Supplementary Information on "Prediction of Heterodimeric Protein Complexes from Weighted Protein-Protein Interaction Networks Using Novel Features and Kernel Functions"

Peiying Ruan, Morihiro Hayashida, Osamu Maruyama and Tatsuya Akutsu

Results on our kernel by another combination

Instead of Eq. (5) in the main manuscript, we examined the combination kernel represented by

$$(1-\beta)K(\phi(C_i),\phi(C_j))+\beta K_c(C_i,C_j), \tag{S1}$$

where β ($0 \leq \beta \leq 1$) is a constant, and specifies, in a percentage, how much the domain composition kernel K_c will factor in. Figure S2 shows the results on the average F-measures using four sets of features and the domain composition kernel with $\beta = 0.0, 0.1 \cdots, 1.0$. We can see that the average F-measures during $0.2 \leq \beta \leq 0.7$ were larger than those of $\beta = 0.0, 1.0$. Table S1 shows the results on the average precision, recall, and F-measure using our combination kernel represented by Eq. (S1) in the best average F-measure case for each set of features of (F1-5), (F1-6), (F1-5,7), and (F1-7).
